# Supplementary material for: Preferential Expansion of HPV16 E1-Specific T Cells from Healthy Donors’ PBMCs after Ex Vivo Immunization with an E1E2E6E7 Fusion Antigen
Source: Cancers (Basel). 2023 Dec 15;15(24):5863. doi: 10.3390/cancers15245863 (PMC10741473; doi:10.3390/cancers15245863)
Supplement: Supplementary file 1 [file cancers-15-05863-s001.zip › cancers-2721320-supplementary.pdf]

Supplementary Materials

Peptide name	HLA	Antigen	Aa	Sequence	Literature (PMID)
E1 aa253-262	A2	E1	253-262	TLLQQYCLYL	9349483
E2 aa69-77	A2	E2	69-77	ALQAIELQL	16425257, 9349483, 30154146
E2 aa138-147	A2	E2	138-147	YICEEASVTV	16425257
E2 aa93-101	A2	E2	93-101	TLQDVSLEV	30154146
E6 aa18-26	A2	E6	18-26	KLPQLCTEL	21550027, 12692247
E6 aa29-38	A2	E6	29-38	TIHDIILECV	17108051
E6 aa52-60	A2	E6	52-60	FAFRDLCIV	30154146, 26390407
E7 aa11-20	A2	E7	11-20	YMLDLQPETT	29603667, 30154146, 26390407, 10999722, 17555571, 10096544, 15639645, 14522932, 21892941, 15963358, 16284959, 16425257, 31964784, 15819706, 12692247, 16107858
E7 aa82-90	A2	E7	82-90	LLMGTLGIV	30154146, 26390407, 17555571, 14522932, 21167863, 21550027
E7 aa86-93	A2	E7	86-93	TLGIVCPI	30154146, 26390407, 10999722, 17555571, 10096544, 14522932, 9748126, 16284959, 16425257, 15819706
E7 aa7-15	A2, B48	E7	7-15	TLHEYMLDL	26390407

Figure S1. Selection of HLA-A2-restricted HPV16 peptides based on existing relevant literature. Selected HLA-A2-restricted HPV16 peptides were used to screen healthy donors' PBMCs to discriminate between HPV16 responders and non-responders, in addition to elucidate the specificity and the breadth of the response.

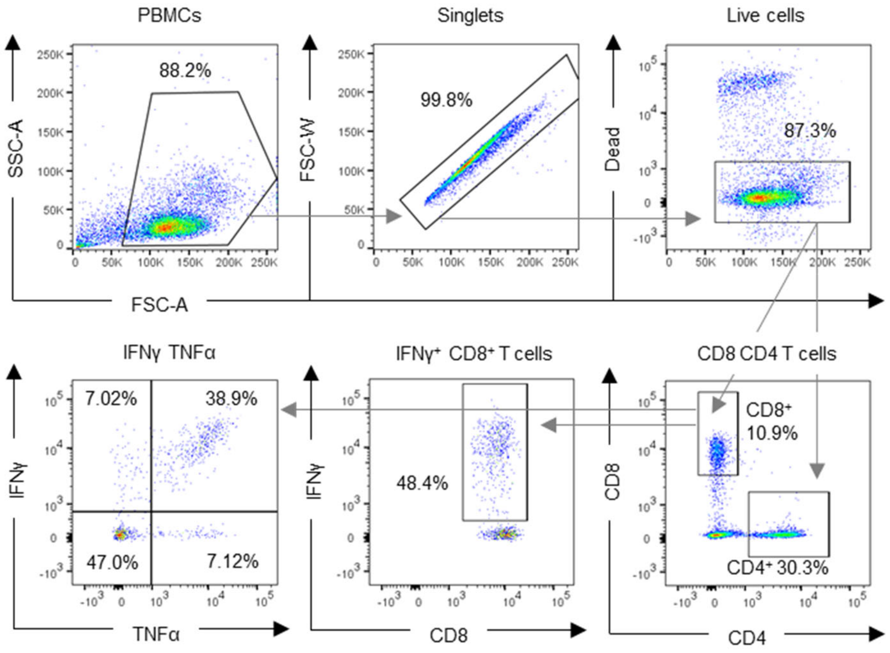

Figure S2. Gating strategy used for flow cytometry data analysis. Representation of the gating strategy used to assess responsiveness of healthy donors to HPV16 (CD8 IFNγ and TNFα secretion) after stimulation of PBMCs from healthy donors with HPV16 HLA-A2 single peptides or HPV16 E1, E2, E6 or E7 peptide pools. These gates applied also for $CD4^+$ T cells.

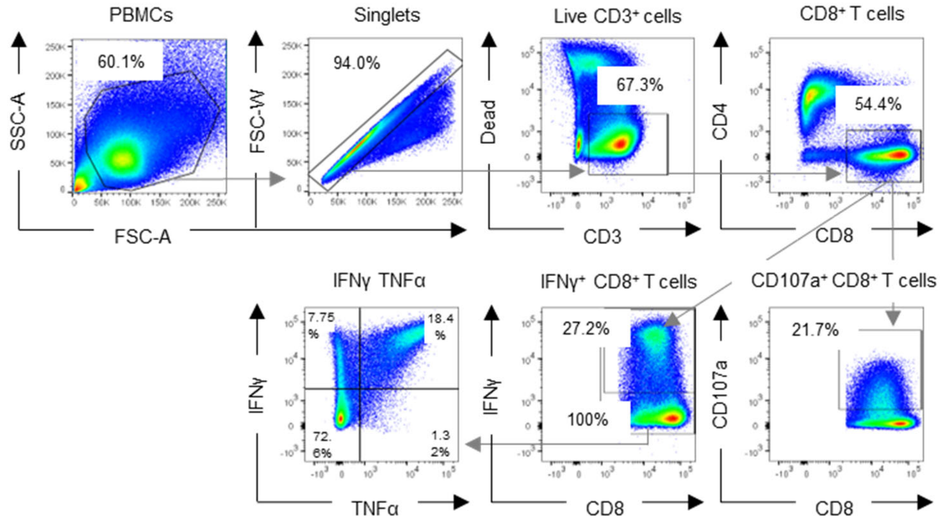

Figure S3. General gating strategy used for flow cytometry data analysis. Representation of the gating strategy used to assess CD8 T cell IFNγ, TNFα and CD107a production after HPV16 HLA-A2 single peptide, HPV16 E1, E2, E6 or E7 peptide pool, or Ca Ski cells stimulation. These gates were also applicable for CD4⁺ T cells.

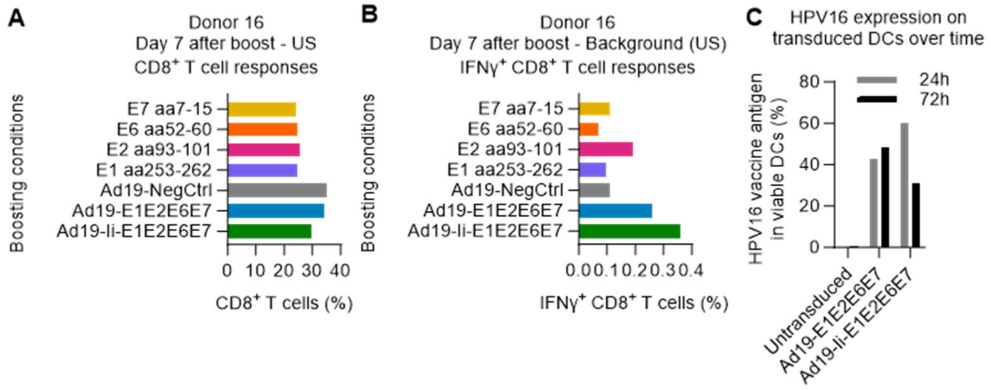

Figure S4. Background comparison of different T cell prime-boost expansion regimens. PBMCs from donor 16 were primed with HPV16 E1 aa253-262, E2 aa93-101, E6 aa52-60 or E7 aa7-15 peptides and boosted two weeks later with either the cognate peptides, or Ad19-transduced DCs without a transgene (Ad19-NegCtrl) or encoding HPV16 with Ii (Ad19-Ii-E1E2E6E7) and without (Ad19-E1E2E6E7). One week later, cells were left unstimulated or were stimulated with HPV16 E1 aa253-262, E2 aa93-101, E6 aa52-60 or E7 aa7-15 peptides and IFNγ$^+$ CD8$^+$ T cell responses were evaluated by flow cytometry. Here, we were specifically looking at the unstimulated samples to evaluate the background responses and therefore choose the most suitable controls. (**A**) Fraction (%) of CD8$^+$ T cells out of the alive CD3$^+$ cells for the different booster regimens showing that Ad19-transduced DCs tended to generate more CD8$^+$ T cells compared to single peptide stimulation. (**B**) Fraction (%) of IFNγ$^+$ in CD8$^+$ T cells for the different booster regimens showing that Ad19-HPV16-transduced DCs, especially when bearing Ii (Ad19-Ii-E1E2E6E7), elicited higher IFNγ$^+$ CD8$^+$ T cell responses when left unstimulated. (**C**) Evaluation of HPV16 vaccine antigen expression 24 and 72 h after Ad19-Ii-E1E2E6E7 or Ad19-E1E2E6E7 transduction showing that the vaccine encoded antigen was still detected 72 h after transduction. This continuous antigen expression upon transduction probably translated into a prolonged T cell stimulation and activation and thus, increased IFNγ production. In contrast, peptide stimulated T cells received a shorter and thus transient activation. Therefore Ad19-NegCtrl (empty vector) was used to subtract the background of T cells stimulated with Ad19-transduced DC in Figure 3 and Figure 4.

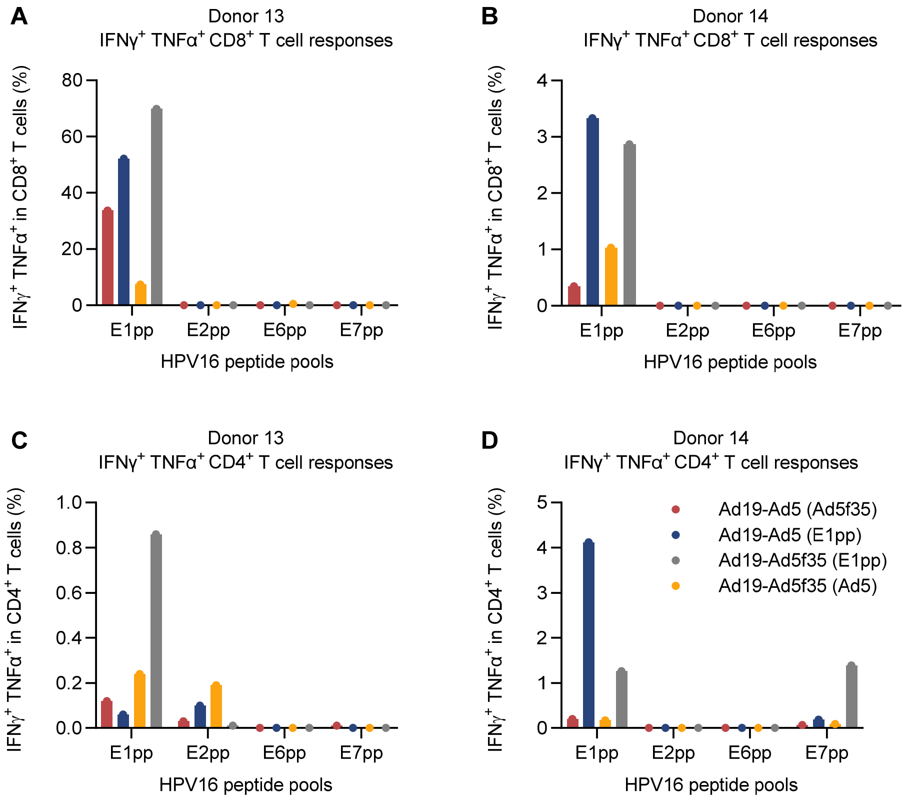

Figure S5. HPV16 T cell responses acquired E1 immunodominance over time. Donor 13 and 14 PBMCs primed with Ad19-Ii-E1E2E6E7-transduced DCs and boosted with either Ad5- or Ad5f35-Ii-E1E2E6E7-transduced DCs were shortly restimulated with either E1 HPV16 peptide pool (E1pp) or with Ad5- or Ad5f35-Ii-E1E2E6E7-transduced DCs (not matching the boost vector) for IFNγ sorting and REP. 15 days after REP, the different effector T cells were restimulated with HPV16 peptide pools (E1pp, E2pp, E6pp or E7pp) and T cells were stained intracellularly for both IFNγ and TNFα and analyzed by flow cytometry. T cells left unstimulated during ICS were used as background and were subtracted from the peptide pool stimulated samples. (**A-B**) Fraction (%) of double positive T cells in $CD8^+$ T cells from donor 13 and 14 showing solely E1-specific reactivity. Interestingly, donor 13 showed strong reactivity against E1 peptide pool stimulation and secreted high amounts of IFNγ and TNFα cytokines. (**C-D**) Fraction (%) of double positive cells in $CD4^+$ T cells from donor 13 and 14. $CD4^+$ T cells showed poor reactivity and secreted low amounts of IFNγ and TNFα in response to E1, E2, and E7.

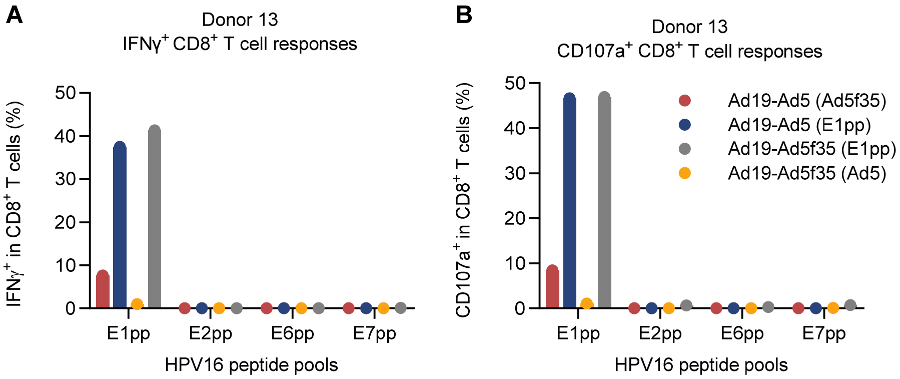

Figure S6. Donor 13 CD8⁺ T cell responses upon stimulation with HPV16 peptide pools. The remaining effector T cells from the ICS and killing assays were frozen. Donor 13 T cells were thawed and after letting them recover for 5 days, they were restimulated with different HPV16 peptide pools or Ca Ski cells. CD8 T cells were surface stained for CD107a and intracellularly stained for IFNγ and analyzed by flow cytometry. (**A**) Fraction (%) of $IFN\gamma^+$ in $CD8^+$ T cells showing retention of E1 immunodominance. (**B**) Fraction (%) of $CD107a^+$ in $CD8^+$ T cells exhibiting degranulation only when stimulated with E1 HPV16 peptide pool.
